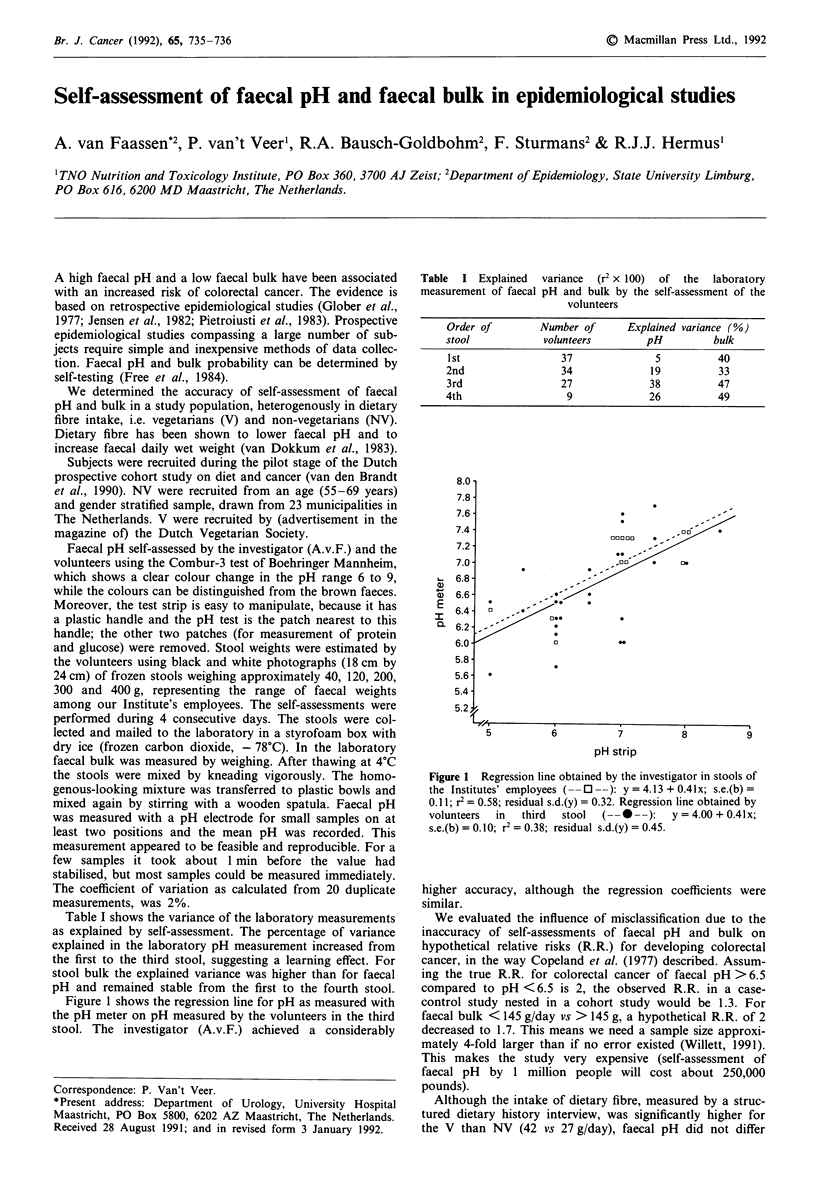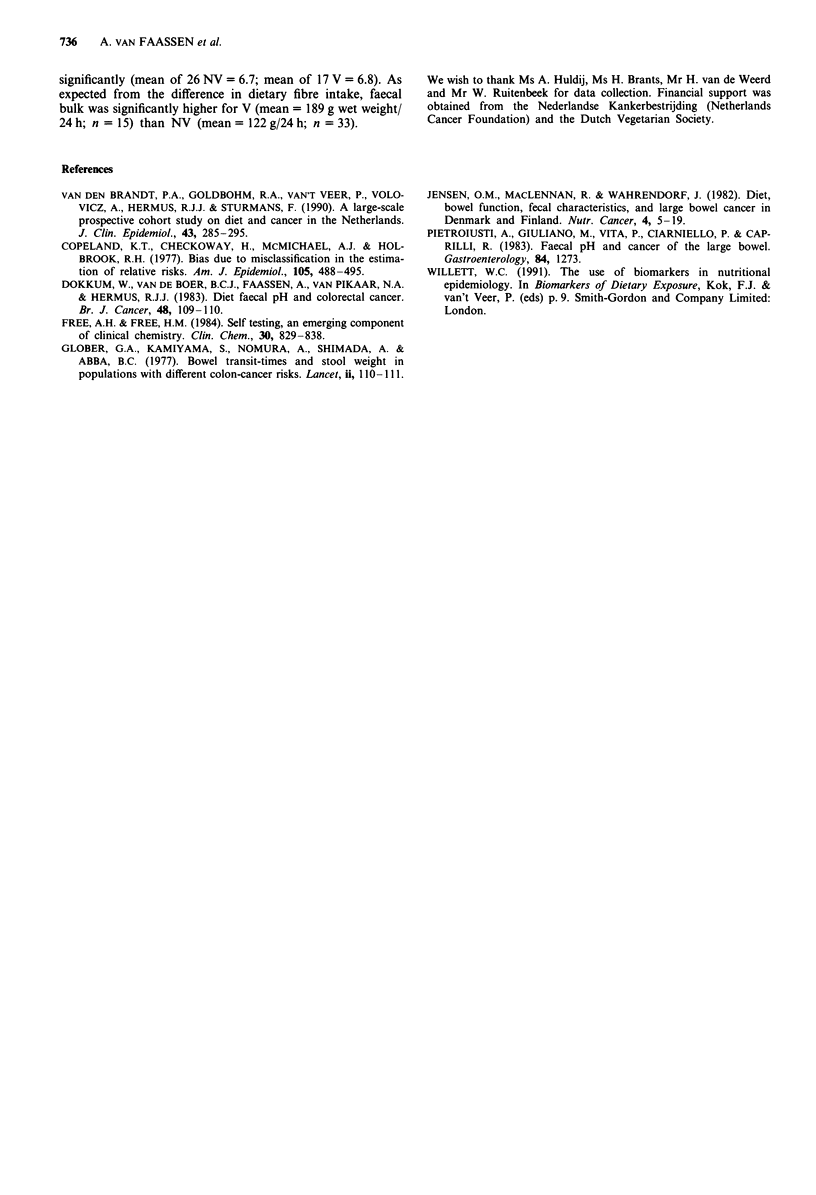# Self-assessment of faecal pH and faecal bulk in epidemiological studies.

**DOI:** 10.1038/bjc.1992.155

**Published:** 1992-05

**Authors:** A. van Faassen, P. van't Veer, R. A. Bausch-Goldbohm, F. Sturmans, R. J. Hermus

**Affiliations:** TNO Nutrition and Toxicology Institute, Zeist, The Netherlands.


Br. J. Cancer (1992), 65, 735–736 © Macmillan Press Ltd., 1992

### Self-assessment of faecal pH and faecal bulk in epidemiological studies

A. van Faassen*[2], P. van't Veer[1], R.A. Bausch-Goldbohm[2], F. Sturmans[2] & R.J.J. Hermus[1]

[1]*TNO Nutrition and Toxicology Institute, PO Box 360, 3700 AJ Zeist;* [2]*Department of Epidemiology, State University Limburg, PO Box 616, 6200 MD Maastricht, The Netherlands.*

A high faecal pH and a low faecal bulk have been associated with an increased risk of colorectal cancer. The evidence is based on retrospective epidemiological studies (Glober *et al.*, 1977; Jensen *et al.*, 1982; Pietroiusti *et al.*, 1983). Prospective epidemiological studies compassing a large number of subjects require simple and inexpensive methods of data collection. Faecal pH and bulk probability can be determined by self-testing (Free *et al.*, 1984).

We determined the accuracy of self-assessment of faecal pH and bulk in a study population, heterogenously in dietary fibre intake, i.e. vegetarians (V) and non-vegetarians (NV). Dietary fibre has been shown to lower faecal pH and to increase faecal daily wet weight (van Dokkum *et al.*, 1983).

Subjects were recruited during the pilot stage of the Dutch prospective cohort study on diet and cancer (van den Brandt *et al.*, 1990). NV were recruited from an age (55–69 years) and gender stratified sample, drawn from 23 municipalities in The Netherlands. V were recruited by (advertisement in the magazine of) the Dutch Vegetarian Society.

Faecal pH self-assessed by the investigator (A.v.F.) and the volunteers using the Combur-3 test of Boehringer Mannheim, which shows a clear colour change in the pH range 6 to 9, while the colours can be distinguished from the brown faeces. Moreover, the test strip is easy to manipulate, because it has a plastic handle and the pH test is the patch nearest to this handle; the other two patches (for measurement of protein and glucose) were removed. Stool weights were estimated by the volunteers using black and white photographs (18 cm by 24 cm) of frozen stools weighing approximately 40, 120, 200, 300 and 400 g, representing the range of faecal weights among our Institute's employees. The self-assessments were performed during 4 consecutive days. The stools were collected and mailed to the laboratory in a styrofoam box with dry ice (frozen carbon dioxide, – 78°C). In the laboratory faecal bulk was measured by weighing. After thawing at 4°C the stools were mixed by kneading vigorously. The homogenous-looking mixture was transferred to plastic bowls and mixed again by stirring with a wooden spatula. Faecal pH was measured with a pH electrode for small samples on at least two positions and the mean pH was recorded. This measurement appeared to be feasible and reproducible. For a few samples it took about 1 min before the value had stabilised, but most samples could be measured immediately. The coefficient of variation as calculated from 20 duplicate measurements, was 2%.

Table I shows the variance of the laboratory measurements as explained by self-assessment. The percentage of variance explained in the laboratory pH measurement increased from the first to the third stool, suggesting a learning effect. For stool bulk the explained variance was higher than for faecal pH and remained stable from the first to the fourth stool.

Figure 1 shows the regression line for pH as measured with the pH meter on pH measured by the volunteers in the third stool. The investigator (A.v.F.) achieved a considerably higher accuracy, although the regression coefficients were similar.

**Table I** Explained variance ($r^2 \times 100$) of the laboratory measurement of faecal pH and bulk by the self-assessment of the volunteers

| *Order of stool* | *Number of volunteers* | *Explained variance (%)* | |
|---|---|---|---|
| | | *pH* | *bulk* |
| 1st | 37 | 5 | 40 |
| 2nd | 34 | 19 | 33 |
| 3rd | 27 | 38 | 47 |
| 4th | 9 | 26 | 49 |

**Figure 1** Regression line obtained by the investigator in stools of the Institutes' employees (--□--): $y = 4.13 + 0.41x$; s.e.(b) = 0.11; $r^2 = 0.58$; residual s.d.(y) = 0.32. Regression line obtained by volunteers in third stool (--●--): $y = 4.00 + 0.41x$; s.e.(b) = 0.10; $r^2 = 0.38$; residual s.d.(y) = 0.45.

We evaluated the influence of misclassification due to the inaccuracy of self-assessments of faecal pH and bulk on hypothetical relative risks (R.R.) for developing colorectal cancer, in the way Copeland *et al.* (1977) described. Assuming the true R.R. for colorectal cancer of faecal pH $>6.5$ compared to pH $<6.5$ is 2, the observed R.R. in a case-control study nested in a cohort study would be 1.3. For faecal bulk $<145$ g/day *vs* $>145$ g, a hypothetical R.R. of 2 decreased to 1.7. This means we need a sample size approximately 4-fold larger than if no error existed (Willett, 1991). This makes the study very expensive (self-assessment of faecal pH by 1 million people will cost about 250,000 pounds).

Although the intake of dietary fibre, measured by a structured dietary history interview, was significantly higher for the V than NV (42 *vs* 27 g/day), faecal pH did not differ

Correspondence: P. Van't Veer.
*Present address: Department of Urology, University Hospital Maastricht, PO Box 5800, 6202 AZ Maastricht, The Netherlands.
Received 28 August 1991; and in revised form 3 January 1992.

significantly (mean of 26 NV = 6.7; mean of 17 V = 6.8). As expected from the difference in dietary fibre intake, faecal bulk was significantly higher for V (mean = 189 g wet weight/24 h; $n$ = 15) than NV (mean = 122 g/24 h; $n$ = 33).

We wish to thank Ms A. Huldij, Ms H. Brants, Mr H. van de Weerd and Mr W. Ruitenbeek for data collection. Financial support was obtained from the Nederlandse Kankerbestrijding (Netherlands Cancer Foundation) and the Dutch Vegetarian Society.

### References

VAN DEN BRANDT, P.A., GOLDBOHM, R.A., VAN'T VEER, P., VOLOVICZ, A., HERMUS, R.J.J. & STURMANS, F. (1990). A large-scale prospective cohort study on diet and cancer in the Netherlands. *J. Clin. Epidemiol.*, **43,** 285–295.

COPELAND, K.T., CHECKOWAY, H., MCMICHAEL, A.J. & HOLBROOK, R.H. (1977). Bias due to misclassification in the estimation of relative risks. *Am. J. Epidemiol.*, **105,** 488–495.

DOKKUM, W., VAN DE BOER, B.C.J., FAASSEN, A., VAN PIKAAR, N.A. & HERMUS, R.J.J. (1983). Diet faecal pH and colorectal cancer. *Br. J. Cancer*, **48,** 109–110.

FREE, A.H. & FREE, H.M. (1984). Self testing, an emerging component of clinical chemistry. *Clin. Chem.*, **30,** 829–838.

GLOBER, G.A., KAMIYAMA, S., NOMURA, A., SHIMADA, A. & ABBA, B.C. (1977). Bowel transit-times and stool weight in populations with different colon-cancer risks. *Lancet*, **ii,** 110–111.

JENSEN, O.M., MACLENNAN, R. & WAHRENDORF, J. (1982). Diet, bowel function, fecal characteristics, and large bowel cancer in Denmark and Finland. *Nutr. Cancer*, **4,** 5–19.

PIETROIUSTI, A., GIULIANO, M., VITA, P., CIARNIELLO, P. & CAPRILLI, R. (1983). Faecal pH and cancer of the large bowel. *Gastroenterology*, **84,** 1273.

WILLETT, W.C. (1991). The use of biomarkers in nutritional epidemiology. In *Biomarkers of Dietary Exposure*, Kok, F.J. & van't Veer, P. (eds) p. 9. Smith-Gordon and Company Limited: London.